# Extensive Dissection at No. 12 Station During D2 Lymphadenectomy Improves Survival for Advanced Lower-Third Gastric Cancer: A Retrospective Study From a Single Center in Southern China

**DOI:** 10.3389/fonc.2021.760963

**Published:** 2022-01-11

**Authors:** Weigang Dai, Er-Tao Zhai, Jianhui Chen, Zhihui Chen, Risheng Zhao, Chuangqi Chen, Yujie Yuan, Hui Wu, Shirong Cai, Yulong He

**Affiliations:** ^1^ Center of Gastrointestinal Surgery, The First Affiliated Hospital, Sun Yat-Sen University, Guangzhou, China; ^2^ Center of Gastric Cancer, Sun Yat-Sen University, Guangzhou, China

**Keywords:** lymphadectomy, advanced gastric cancer, hepatoduodenal ligament, oncological outcomes, complications

## Abstract

**Background:**

D2 lymphadenectomy including No. 12a dissection has been accepted as a standard surgical management of advanced lower-third gastric cancer (GC). The necessity of extensive No. 12 nodes (No. 12a, 12b, and 12p) dissection remains controversial. This study aims to explore its impact on long-term survival for resectable GC.

**Methods:**

From 2009 to 2016, 353 advanced lower-third GC patients undergoing at least D2 lymphadenectomy during a radical surgery were included, with 179 patients receiving No. 12a, 12b, and 12p dissection as study group. A total of 174 patients with No. 12a dissection were employed as control group. Surgical and long-term outcomes including 90-day complications incidence, therapeutic value index (TVI), 3-year progression-free survival (PFS), and 5-year overall survival (OS) were compared between both groups.

**Results:**

No. 12 lymph node metastasis was observed in 20 (5.7%) patients, with 10 cases in each group (5.6% vs. 5.7%, *p *= 0.948). The metastatic rates at No. 12a, 12b, and 12p were 5.7%, 2.2%, and 1.7%, respectively. The incidence of 90-day complications was identical between both groups. Extensive No. 12 dissection was associated with increased TVI at No. 12 station (3.9 vs. 0.6), prolonged 3-year PFS rate (67.0% vs. 55.9%, *p* = 0.045) and 5-year OS rate (66.2% vs. 54.0%, *p *= 0.027). The further Cox-regression analysis showed that the 12abp dissection was an independent prognostic factor of improved survival (*p *= 0.026).

**Conclusion:**

Adding No. 12b and 12p lymph nodes to D2 lymphadenectomy might be effective in surgical treatment of advanced lower-third GC and improve oncological outcomes compared with No. 12a-based D2 lymphadenectomy.

**Graphical Abstract:**
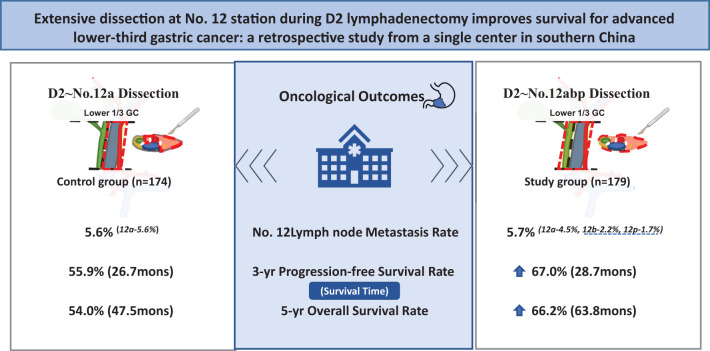


## Introduction

Gastric cancer (GC) is the second most common malignance disease worldwide, and gastric adenocarcinoma accounts for approximately 95% of gastric cancer (1). Despite of recent progress in the treatment of gastric adenocarcinoma, the outcome is still poor with a 5-year overall survival (OS) rate of about 20% (2). Surgical resection with adequate lymph node dissection remains the curable therapy for such disease. Nevertheless, over 50% of patients present with locally advanced or metastatic gastric adenocarcinoma at first diagnosis, with chemotherapy or immunotherapy as the main therapeutic regimen for those patients (1, 3). Lymph node metastasis is one of the most important determinants of long-term outcomes in patients with advanced GC. The GC treatment guideline from Japanese Gastric Cancer Association recommends dissection of all first- and second-tier lymph nodes (D2) with gastrectomy as standard therapy for advanced GC in Japan (4).

The hepatoduodenal lymph nodes, known as the No. 12 lymph nodes, can be divided into three groups of nodes along the hepatic artery (No. 12a), the bile duct (No. 12b), and behind the portal vein (No. 12p). Previously, the No. 12b and 12p lymph nodes were regarded as third-tier (N3) lymph nodes. Unfortunately, the data of No. 12b and 12p lymph nodes were limited and mostly available from Japan and South Korea. Feng et al. (5) reported that the metastatic rates of No. 12b and 12p lymph nodes were 3.1% and 9.2%, respectively. The necessity of adding No. 12b and 12p dissection into D2 lymphadenectomy remains an unsolved problem in GC surgery. In China, most of GC patients were diagnosed at advanced stage, with no more than 10% early stage observed (6, 7). To fully investigate the oncological outcomes of No. 12 lymph node dissection, we retrospectively compared the short- and long-term outcomes of an extensive No. 12 dissection (No. 12abp) with No. 12a dissection alone for patients with resectable GC.

## Methods

### Patients

**Figure 1:**
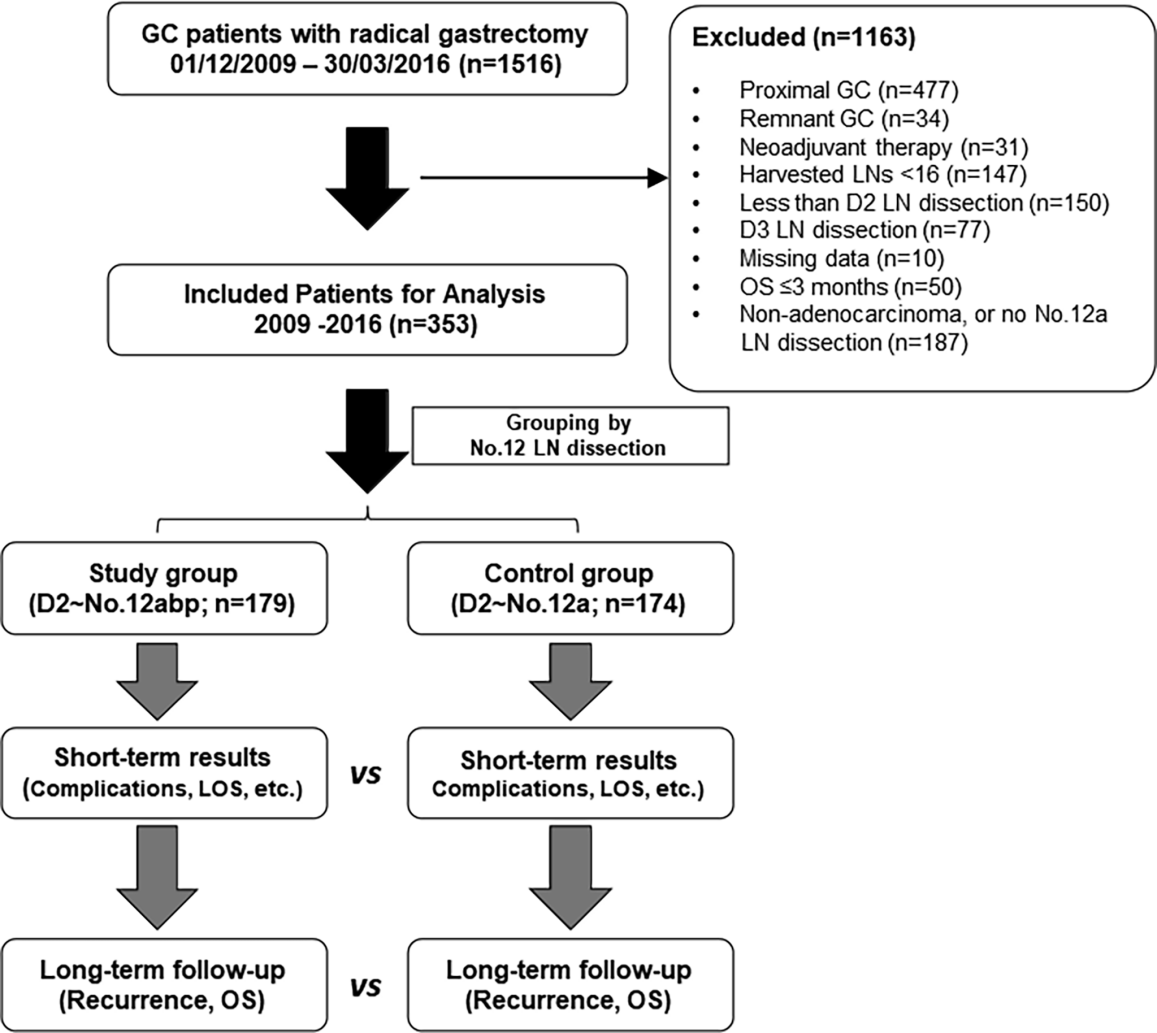
The flowchart of inclusion and exclusion criteria in this study. Both short- and long-term outcomes were compared between the two groups. GC, gastric cancer; LN, lymph node; LOS, length of stay; vs, versus; OS, overall survival.

This is a single-center, retrospective study for oncological outcomes of advanced GC. From December 2009 to March 2016, patients with resectable GC who underwent a radical gastrectomy at our unit were first enrolled. The including criteria were as follows (1): confirmed diagnosis of gastric adenocarcinoma, which was restricted to the lower third of stomach and resectable based on a multidisciplinary team (MDT) discussion (8) and (2) underwent D2 lymphadenectomy with at least No. 12a lymph nodes dissected. The flow chart and exclusion criteria of the current study are shown in **Figure 1**. The study protocol was approved by the Ethics Committee of the First Affiliated Hospital of Sun Yat-Sen University. All patients had signed an informed consent form for either surgery or chemotherapy. All processes involved in this study were in accordance with the ethics statements presented in the 1964 Declaration of Helsinki and its later amendments.

Preoperative tumor staging was performed by means of contrast-enhanced computed tomography (CT) scans or endoscopic ultrasonography, and surgical plan for each patient was mainly decided through a routine MDT meeting at fixed time (9). In brief, clinical TNM stages from T2N0M0 to T4N2M0 were allowed a radical tumor resection through open or laparoscopic surgery in our unit. Patients were divided into two groups based on whether an extended lymphadenectomy at No. 12 station performed or not: control group, including those undergoing standard D2 lymphadenectomy with No. 12a dissection alone; and study group, composed of additional 12b and 12p node dissection to D2 procedure (**Supplementary Figure S1**). In our center, we treated No. 12a LN metastasis as a type of regional metastasis from a primary gastric cancer; therefore, we routinely dissected No. 12a LNs during the D2 lymphadenectomy.

### Surgical Approaches for No. 12 Lymph Node Dissection

**Figure 2:**
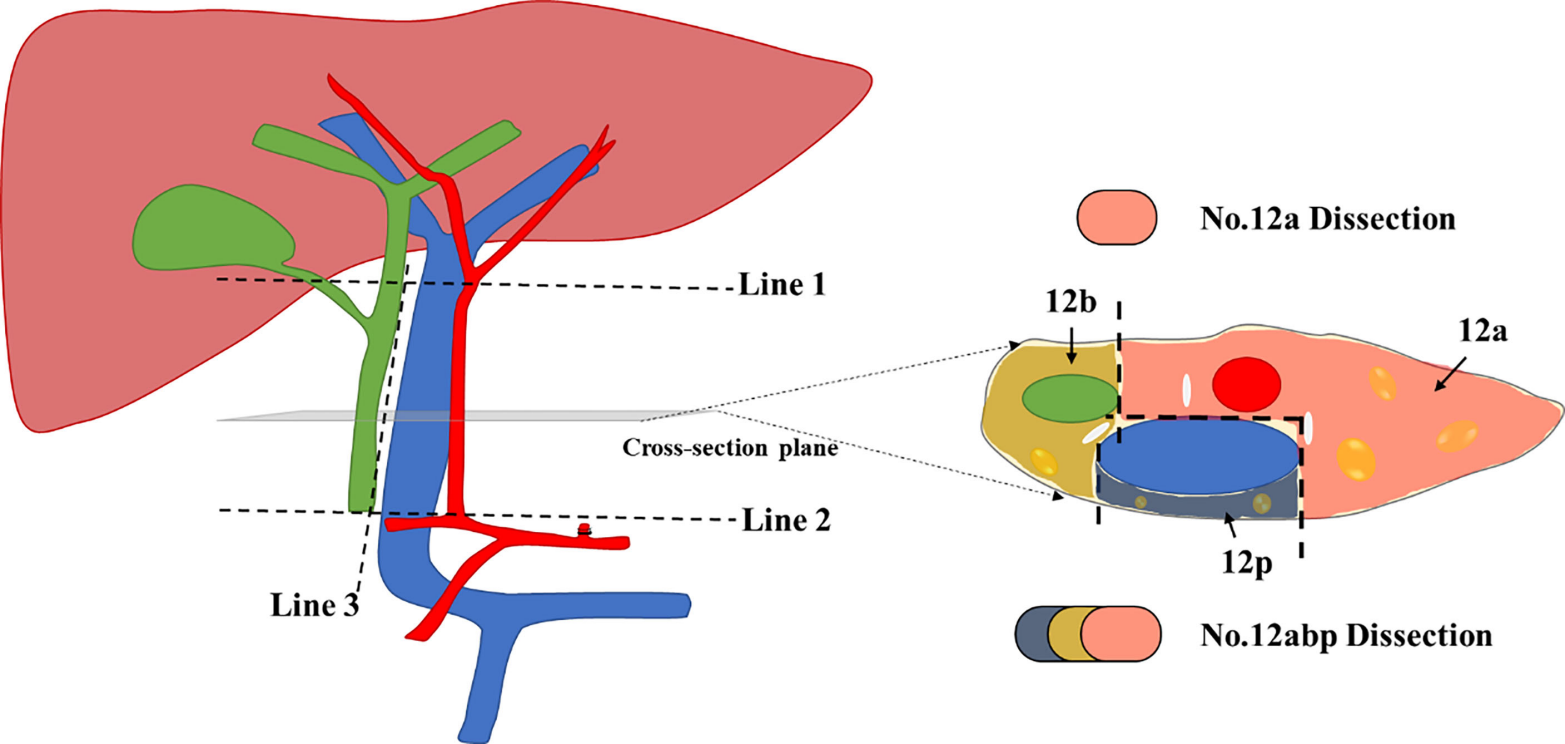
The modified scope definition of No. 12 Lymph nodes during lymphadenectomy for GC. Line 1 indicates the upper border of No. 12 lymph nodes (LNs), which is level-up the confluence of the left and right hepatic artery. Line 2 indicates the lower border of No. 12 LNs, which is level-up the origin of the proper hepatic artery. Line 3 indicates the left offside border of the common bile duct; The cross-section diagram of hepatoduodenal ligament reveals the separated zones for 12a, 12b, and 12p LNs, respectively. We used three distinct colors to mark each area for harvested LNs during No. 12 lymphadenectomy.

Given that the scope definition of No. 12a, 12b, and 12p LNs is not clearly defined according to the guidelines of American Joint Committee on Cancer (AJCC)/Union for International Cancer Control or Japanese treatment guidelines, we utilized a peer consensus which was capable to describe No. 12 LN dissection during operation (10). The modified illustration of such scope definition of No. 12a, 12b, and 12p is presented as **Figure 2**, similar to published demonstration (10, 11).

The surgical procedures of No. 12 lymph node dissection were performed as previously described (10). In short, hepatoduodenal ligament was fully exposed from the first part of duodenum to the lower border of the right liver. Afterward, the ligament was opened from the left borderline of common bile duct (Line 3, **Figure 2**) until the bifurcation of the left and right hepatic arteries (Line 1, **Figure 2**). The perivascular sheathes along the proper hepatic artery and portal vein were fully opened, with retraction bands utilized to protect vital vessels during the No. 12 lymphadenectomy. All tissues were then harvested from three different cross-section zones (**Figure 2**), with isolated LNs immediately stored in a station-marked container to avoid confusion with the other harvested tissues during the surgery.

Also, the detailed gastrectomy and reconstruction was mainly dictated by surgeon’s preference, with open or laparoscopic fashion employed across the study period. All surgeries were completed by over 10-year experienced gastrointestinal surgeons in our center. As for adjuvant chemotherapy, fluorouracil (5-FU)-based chemotherapy was scheduled for patients with stage II or higher, with combining S-1 (60 mg/m^2^) plus oxaliplatin (85 mg/m^2^) as a main regimen for at least eight cycles in planning. Neoadjuvant therapy was not included in the current study.

### Follow-Up

The postoperative follow-up of the patients was recorded by the research nurse of our center, as previously described (12). Typically, follow-up visits at outpatient clinics were scheduled every 3 months for the first 2 years after surgery, every 6 months for the next 3 years, and then once a year or until death. Physical examination and laboratory tests (including tumor markers) were required at each visit, while chest and abdominal CT scans with enhanced contrast were performed every 6 months or annually. Gastric endoscopy was performed every year. Tumor recurrence was mainly confirmed based on clinical, radiological, or endoscopic signs of disease. Laparoscopic exploration and biopsy were applied only in cases enrolling in clinical trials. Recurrence and survival status was last updated on March 31, 2021.

### Study Outcomes

The incidence of 90-day complications after radical surgery was utilized to evaluate the safety of No. 12 lymphadenectomy. The severity of complications was evaluated with the Clavien-Dindo classification (13, 14). The 3-year progression-free survival (PFS) and 5-year overall survival (OS) were employed to explore oncologic outcomes for included patients. Briefly, PFS was calculated from the time of operation until evidence of disease progression or recurrence, a new primary cancer, or cancer-related death, while OS was calculated from the time of operation to the time of death or final follow-up.

As previously mentioned (15, 16), the therapeutic value index (TVI) was employed to evaluate the impact of lymphadenectomy on long-term survival, which was calculated by multiplying the metastatic rate of lymph node by the 5-year OS rate of the patients with metastasis to that station. The incidence of lymph node metastasis, also noticed as frequency of lymph node metastasis, was calculated by dividing the number of patients with metastasis at the given lymph node station by the number of patients in whom that station was dissected.

**TVI (N) **=** **Lymph node metastatic rate (N)** **×** **5-year OS rate (N)** **×** **100 (N for node station)

Additionally, the ratio of lymph node metastasis, calculated by the number of positive nodes divided by the total harvested number of nodes at the given station, was employed to describe the severity of lymph node metastasis in enrolled patients.

### Statistical Analysis

Descriptive statistics were used to present demographic and oncologic features of included patients. Quantitative variables were expressed as means ± standard deviation (SD) or median with range, with categorical variables described as frequency plus percentages. The continuous variables were analyzed by means of the Student’s *t*-test, while the categorical variables were analyzed using the *Chi*-square or *Fisher* exact test. The OS curves were calculated using the Kaplan-Meier method based on the duration of time between the primary surgical treatment and the final follow-up or death. The Log-rank test was used to evaluate the significant differences between curves. The Cox proportional hazards regression model was implemented to determine the independent prognostic factors of long-term survival. The statistical analysis was accomplished by using the statistical analysis program package for Social Science software (SPSS, version 23, IBM^®^, Chicago, IL, USA). Two-tailed tests were applied, with *p* ****<** **0.05 considered statistically significant.

## Results

### Patient Characteristics

Within the study period (2009–2016), 1,516 GC patients underwent radical gastrectomy for GC at our center. Of these, 353 patients were recruited for final analysis: 174 patients had received No. 12a node dissection (control group; *n* ****=** **174) and 179 patients had undergone No. 12abp node dissection (study group; *n* ****=** **179). The baseline and demographic features of included patients are summarized in **Table 1**, without significant difference observed between both groups. Of note, 15 patients were classified into pathological IB stage according to the 7th edition of the TNM classification of GC.

**Table 1:** The baseline and demographic characteristics of patients with gastric cancer.

	Control group (*n* = 174)	Study group (*n* = 179)	*p*-value
	D2 with 12a	D2 with 12a +12b, 12p	
Gender [*n* (%)]			
Mal	105 (60.3)	116 (64.8)	0.387
Female	69 (39.7)	63 (35.2)	
Race (*n* (%))			
Han	174 (100)	179 (100)	NA
Age (years)	56.5** **±** **11.6	55.8** **±** **10.8	0.570
Borrmann type (*n* (%))			
I	4 (2.3)	3 (1.7)	0.902
II	30 (17.2)	35 (19.6)	
III	123 (70.7)	129 (72.1)	
IV	17 (9.8)	12 (6.7)	
Tumor diameter (*n* (%))			
<5 cm	109 (62.6)	129 (72.1)	0.059
≥5 cm	65 (37.4)	50 (27.9)	
Comorbidity (*n* (%))			
DM	12 (6.9)	11 (6.1)	0.775
HTN	28 (16.1)	30 (16.8)	0.866
HD	6 (3.4)	10 (5.6)	0.334
COPD	10 (5.7)	10 (5.6)	0.948
Histology (*n* (%))			
Differentiated	44 (25.3)	44 (24.5)	0.902
Undifferentiated	130 (74.7)	135 (75.5)	
Depth of invasion (*n* (%))			
pT1	9 (5.2)	12 (6.7)	0.447
pT2	16 (9.2)	13 (7.3)	
pT3	44 (25.3)	57 (31.8)	
pT4	105 (60.3)	97 (54.2)	
N stage (*n* (%))			
pN0	37 (21.3)	31 (17.3)	0.094
pN1	43 (24.7)	48 (26.8)	
pN2	35 (20.1)	54 (30.2)	
pN3	59 (33.9)	46 (25.7)	
Pathological TNM stage (*n* (%))			
I	7 (4.0)	8 (4.5)	0.900
II	57 (32.8)	62 (34.6)	
III	110 (63.2)	109 (60.9)	
Preoperative tumor marker (*n* (%))			
CEA ≤5 g/L	144 (82.8)	157 (87.7)	0.189
CA199 ≤35 U/ml	148 (85.1)	156 (87.2)	0.570
Postoperative chemotherapy (*n* (%))			
Yes	118 (67.8)	126 (70.4)	0.601
No	56 (32.2)	53 (29.6)	

DM, diabetes mellitus; HTN, hypertension; HD, heart disease; COPD, chronic obstructive pulmonary disease; NA, not available.

### Surgical and Short-Term Outcomes

The profile of operative procedure and perioperative outcomes are summarized in **Table 2**. Comparing with the control group, laparoscopic operation was less frequently performed, operative duration was markedly extended, and harvested number of lymph nodes in total and No. 12 station were significantly increased in the study group (*p* ****<** **0.05). Other parameters, such as intraoperative blood loss, lymph node metastasis rate for No. 12, and length of stay (LOS) in hospital or after surgery, were comparable between both groups (*p* ****>** **0.05).

**Table 2:** The perioperative outcomes between the two groups.

	Control group (*n* = 174)	Study group (*n* = 179)	*p*-value
	*D2 with 12a*	*D2 with 12a +12b, 12p*	
Surgical approach (*n* (%))			
Open surgery	150 (86.2)	173 (96.6)	0.001
Laparoscopic surgery	24 (13.8)	6 (3.4)	
Operative duration (min)	272.3 ± 67.6	286.6 ± 63.9	0.042
Bleeding volume (ml)	172.2 ± 160.2	159.0 ± 151.9	0.426
Blood transfusion (ml)	158.9 ± 295.5	89.0 ± 210.6	0.010
Harvested LNs (*n* (M, range))	32.0 (10–91)	36.0 (10–93)	0.014
Positive LNs (*n* (M, range))	4.0 (0–43)	3.0 (0–48)	0.591
Harvested No. 12 LNs (*n*)	1.0 (0–11)	4.0 (0–18)	0.001
No. 12 LN metastasis (*n* (%))	10 (5.7%)	10 (5.6%)	0.948
Combined organ resection (*n* (%))	29 (16.7%)	30 (16.8%	0.981
Types of resection (*n* (%))			
Distal gastrectomy	136 (78.2)	147 (82.1)	0.351
Total gastrectomy	38 (21.8)	32 (17.9)	
LOS (day)	25.4 ± 11.4	24.3 ± 10.9	0.355
PLOS (day)	11.1 ± 6.6	10.3 ± 6.5	0.266

M, median; LN, lymph node; LOS, length of stay; PLOS, postoperative length of stay.

To evaluate the safety of aggressive lymph node dissection at No. 12 station, we explored the incidence of 90-day complications after surgery (**Table 3**). There was no significant difference in postoperative complications between the two groups (*p* ****>** **0.05). Specifically, the incidence of biliary leakage or portal vein injury was not recorded in both groups. Of note, the excluded patients (*n* ****=** **50, **Figure 1**) due to survival of less than 3 months did not die from severe complications after surgery. Three patients suffered from unplanned readmission, with one case for delayed intra-abdominal hemorrhage and two cases for wound infections.

**Table 3:** The 90-day postoperative complications for GC patients undergoing radical gastrectomy.

	Control group (*n* = 174)	Study group (*n* = 179)	*p*-value
	*D2 with 12a*	*D2 with 12a +12b, 12p*	
SSI (*n* (%))	19 (10.9)	15 (8.4)	0.419
Superficial	8 (4.6)	4 (2.2)	
Deep	15 (8.6)	10 (5.6)	
Organ or space	1 (0.6)	0 (0)	
Anastomotic leakage (*n* (%))	1 (0.6)	0 (0)	0.493
Duodenal stump leakage (*n* (%))	2 (1.1)	2 (1.1)	1.000
Anastomotic stenosis (*n* (%))	2 (1.1)	0 (0)	0.242
Bowel obstruction	3 (1.7)	2 (1.1)	0.681
Pneumonia	9 (5.2)	10 (5.6)	0.863
Intra-abdominal hemorrhage	2 (1.1)	1 (0.6)	0.619
Biliary leakage	0 (0)	0 (0)	NA
Pancreatic leakage	4 (2.3)	3 (1.7)	0.720
Lymphatic leakage	0 (0)	1 (0.6)	1.000
CVCRI	2 (1.1)	0 (0)	0.242
Unplanned secondary laparotomy	3 (1.7)	0 (0)	0.119
Unplanned 30-day readmission	1 (0.6)	2 (1.1)	1.000
Severity of complications (Clavien-Dindo classification)			
Grade I	1 (0.6)	0 (0)	0.484
Grade II	9 (5.2)	8 (4.5)	
Grade III	15 (8.6)	10 (5.6)	

GC, gastric cancer; SSI, surgical site infection; CVCRI, central venous catheter-related infection; NA, not available.

**Figure 3:**
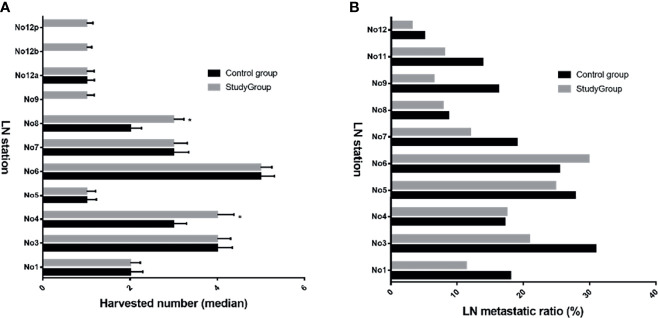
Harvested LNs and metastatic ratio at N1 and N2 stations between both groups. The median harvested number in study group was increased for No. 4, 8, 9, 12b, and 12p **(A)**, ^*^ *p* < 0.05 vs. control group; However, most ratios of metastatic lymph nodes in study group were reduced compared with those in the control group **(B)**. LN, lymph node.

In this study, both harvested and metastatic lymph nodes at N1 and N2 stations for GC were compared between the two groups (**Figure 3**). The overall lymph node metastatic ratios for the control and study groups were 20.4% and 16.0%, respectively. The detailed numbers of harvested and metastatic lymph nodes at each station are summarized in **Supplementary Table S1**. Of note, the metastatic rates at No. 12a, 12b, and 12p stations were 5.7%, 2.2%, and 1.7%, respectively.

### Long-Term Outcomes

We compared TVI values at each station between the two groups and found that most of TVI values in the study group were higher than those in the control group, especially for No. 6 (25.2 vs. 16.7), No. 7 (17.3 vs. 8.0), and No. 12 (3.9 vs. 0.6) values (**Table 4**). Moreover, the cumulative TVI value at all dissected stations was markedly increased in the study group compared with the control group (121.2 vs. 93.6).

**Table 4:** Therapeutic value index of metastatic lymph node station for gastric cancer.

LN station	Control group			Study group		
	Metastatic rate (%)	5-year survival rate (%)	TVI	Metastatic rate (%)	5-year survival rate (%)	TVI
No. 1	20.1	40.0	8.0	11.2	50.0	5.6
No. 3	46.6	44.4	20.7	43.6	56.4	24.6
No. 4	24.7	46.5	11.5	28.5	56.9	16.2
No. 5	33.9	52.5	17.8	25.7	54.3	14.0
No. 6	43.7	38.2	16.7	49.8	50.6	25.2
No. 7	21.8	36.8	8.0	28.5	60.8	17.3
No. 8a	16.1	39.3	6.3	15.6	39.3	6.1
No. 9	9.8	41.2	4.0	6.7	33.3	2.2
No. 11p	4.0	0	0	6.1	45.5	2.8
No. 12a	5.7	10.0	0.6	4.5	87.5	3.9
No. 12b	0	0	0	2.2	100.0	2.2
No. 12p	0	0	0	1.7	66.7	1.1

The value of TVI at No. 12 in the study group calculated as 5.6 multiplied by 70.0%. LN, lymph node; TVI, therapeutic value index.

**Figure 4:**
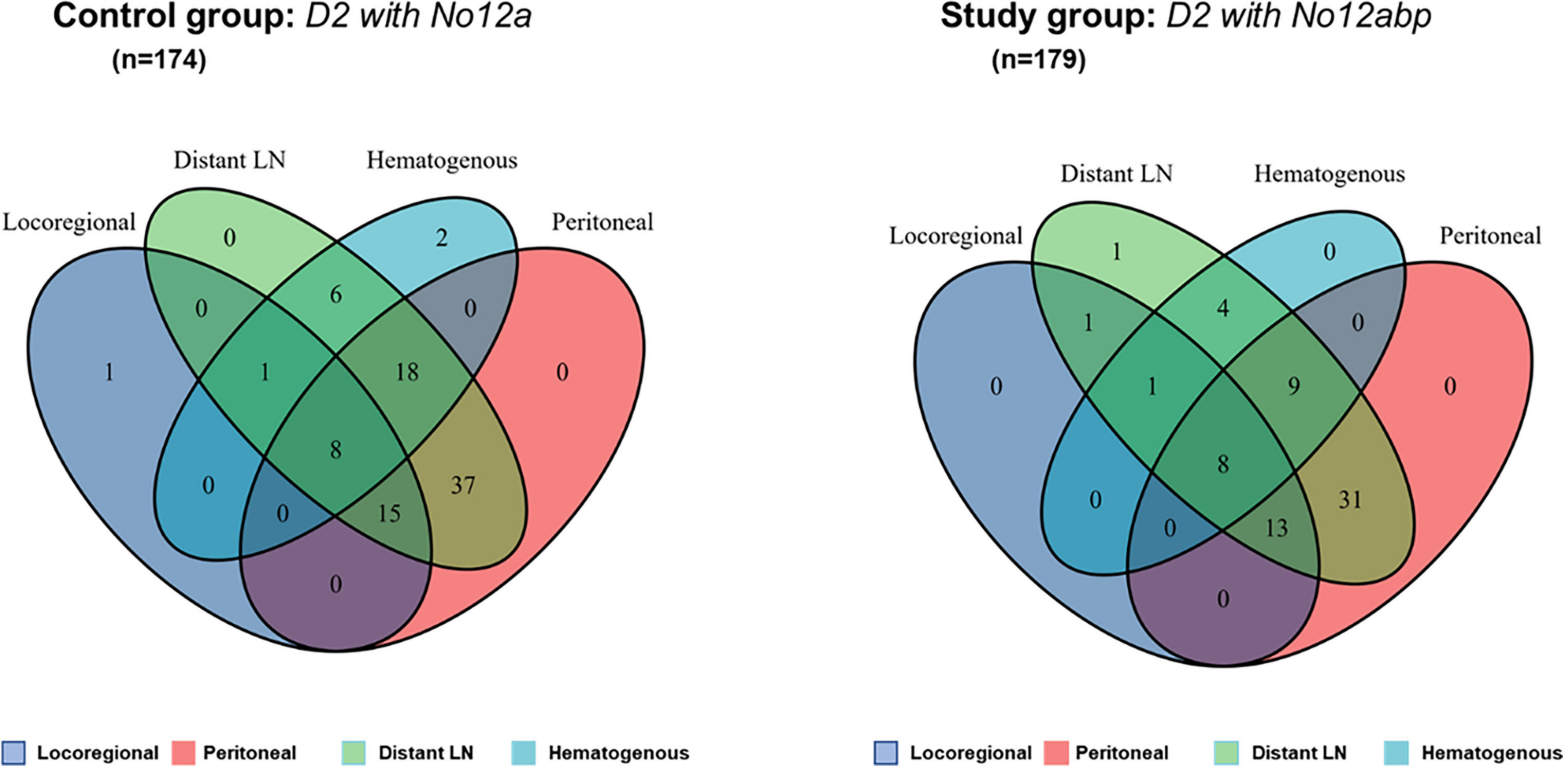
The Venn diagram comparison of recurrence patterns after curative surgery for GC. Numbers in such areas of diagram indicate the number of patients with four types of tumor recurrence. Both diagrams (left and right) were produced by using a public shared tool from Sangerbox.com.

Within a median follow-up of 65.0 months, we have detected recurrence after curative surgery in 156 patients. Among those patients, 88 (55.1%) patients underwent D2 with No. 12a lymphadenectomy during surgery, with 68 (44.9%) patients receiving D2 with No. 12abp procedures. We compared the difference in recurrence patterns between both groups through the Venn diagram (**Figure 4**). The diagram shows that aggressive lymph nodes dissection at No. 12 station has a limited role in reducing distant lymph node metastasis for GC. However, the detailed comparisons (**Supplementary Table S2**) indicate that D2 with No. 12abp node dissection markedly improved hematogenous (*p* = 0.029) and peritoneal (*p* = 0.039) recurrence in such GC patients.

**Figure 5:**
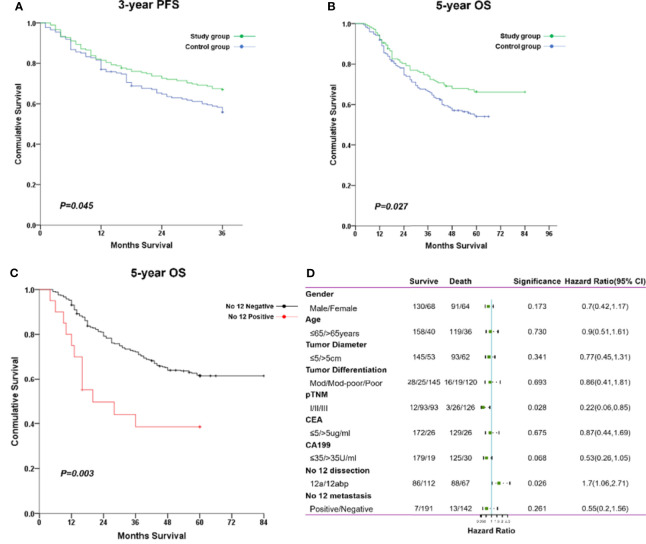
The comparisons of long-term survival between both groups. **(A)** The cumulative rate for 3-year PFS. **(B)** The cumulative rate for 5-year OS. **(C)** The cumulative rate for 5-year OS stratified with No. 12 nodal status. **(D)** The forest plot of factors impacting on overall survival through a multivariate cox regression analysis.

As compared with the control group, the 3-year PFS rate was significantly increased in the study group (67.0% vs. 55.9%, *p* *=* 0.045; **Figure 5A**). The average survival time for PFS was significantly longer in the study group (28.7 months; 95% CI, 26.9–30.4) than in the control group (26.7 months; 95% CI, 24.8–28.6). Also, the 5-year OS rate was markedly improved in the study group (66.2% vs. 54.0%, *p* = 0.027; **Figure 5B**). The average overall survival time for OS was also prolonged in the study group (63.8 months; 95% CI, 59.5–68.2) than in the control group (47.5 months; 95% CI, 44.2–50.8). Of note, metastasis of No. 12 lymph nodes was associated with poor 5-year OS compared with nonmetastasis at such station (**Figure 5C**). Furthermore, the Cox-regression analysis showed that pathological TNM stage (*p* *=* 0.028) and No. 12 LN dissection (*p* *=* 0.026) were independent factors of predicting prognosis of gastric cancer in such populations (**Figure 5D**).

## Discussion

In this study, patients with lower-third GC were included to explore the impact of adding No. 12b and 12p dissection to D2 lymphadenectomy on long-term outcomes in a single cancer center. This extended lymphadenectomy at No. 12 station revealed the metastatic rates of 2.2% and 1.7% for No. 12b and 12p, respectively. Importantly, it was associated with increased TVI at No. 12 station (3.9 vs. 0.6), prolonged both 3-year PFS rate (67.0% vs. 55.9%, *p* = 0.045) and 5-year OS rate (66.2% vs. 54.0%, *p* = 0.027). Moreover, it was regarded as an independent prognostic factor of improved survival (*p* = 0.026) by further Cox-regression analysis. All findings suggested that lymph nodes at No. 12b and 12p stations should be dissected during D2 lymphadenectomy for resectable lower-third GC patients.

Until now, the therapeutic efficacy of lymphadenectomy at each station was rarely evaluated, with limited data available (16). The therapeutic value index was a practical tool to deduce the actual benefit of node dissection without considering the stage migration of GC (15). We employed this novel tool to compare the efficacy of extensive No. 12 nodes dissection to standard D2 procedure. The TVI values at various tier lymph nodes were comparable with similar studies, ranging from 0 to 30.9 (16, 17). Furthermore, the value for extensive No. 12 station dissection was increased compared with No. 12a dissection alone (3.9 vs. 0.6), which suggested that additional No. 12b and 12p nodes dissection yields survival benefits for lower-third GC patients.

In anatomy, the No. 12 lymph nodes are located in the hepatoduodenal ligament, which has been regarded as the free border or the root of ventral mesogastrium (18). Therefore, it is reasonable to regard the No. 12b and 12p lymph nodes as N3 lymph nodes. In clinical practice, the No. 12 lymph nodes were minor stations but often became enlarged leading to bile duct obstruction in advanced GC, especially for lower third GC. As for lower 1/3 GC, the reported rate for No. 12 lymph node metastasis ranged from 4.7% to 11.6% (5, 19–21). Interestingly, the metastasis of No. 12b and 12p lymph nodes were often secondary to No. 5 and No. 12a lymph node metastasis. In our study, the metastatic rates of No. 12b and 12p nodes were 2.2% and 1.7%, respectively. Dissection of No. 12b and 12p lymph nodes had additional improvements in both the 3-year PFS rate and the 5-year OS rate. Multivariate regression analysis confirmed that adding such nodes to D2 lymphadenectomy could be an independent prognostic factor, which was not identical to previous study (5).

Even so, the shortcomings of this study should be mentioned here. First, the nature of a single-center retrospective study design determined the limitation of generating our findings to a more generalized population. Second, the relatively small sample size definitely compromised the power of our findings and brought certain uncertainty to the conclusion. Third, the data about the No. 14v lymph nodes were limited and failed to summarize here. Until now, its dissection depends on the preference of surgeons, with no consensus achieved yet. Several studies found that dissection of No. 14v is related to improved survival in lower-third GC, especially for patients with No. 14v metastasis (22, 23). Last, it could not overcome selecting bias and surgeon’s preference due to the nature of retrospective analysis. The LN metastatic rate was higher in the control group than in the study group, although most values of TVI were improved through an extensive No. 12 LN dissection. Therefore, multicenter randomized controlled trials are indispensable to further validate the current findings.

In conclusion, an extensive dissection of hepatoduodenal ligament lymph nodes, including No. 12a, 12b, and 12p, was safe to apply in the management of resectable lower-third GC. As compared with standardized D2 dissection with No. 12a alone, the extensive lymphadenectomy was associated with increased therapeutic value index and improved long-term outcomes. All findings supported its clinical usage for surgical treatment of advanced lower-third gastric cancer, particularly when curative operation is possible, to improve survival in such populations.

## Data Availability Statement

The raw data supporting the conclusions of this article will be made available by the authors, without undue reservation.

## Ethics Statement

The studies involving human participants were reviewed and approved by the Ethics Committee of the First Affiliated Hospital of Sun Yat-Sen University. The patients/participants provided their written informed consent to participate in this study.

## Author Contributions

SC, HW, and YY conceived and designed the study. YY, WD, and E-TZ collected all included cases and drafted the paper. JC, ZC, and RZ performed data analyses and necessary revision of final manuscript. HW, YH, CC, and SC were responsible for the interpretation of all data and critical revision. All authors read and approved the final manuscript.

## Funding

This study was supported by grants from the National Natural Science Foundation of China (No. 81702878, No. 82003112, and No. 81372341), Guangdong Basic and Applied Basic Research Foundation (2020A1515010214 and 2021A1515010473), Project 5010 of Sun Yat-Sen University (2018004), and Young Teacher Training Project of First Affiliated Hospital of Sun Yat-Sen University (19ykpy58).

## Conflict of Interest

The authors declare that the research was conducted in the absence of any commercial or financial relationships that could be construed as a potential conflict of interest.

## Publisher’s Note

All claims expressed in this article are solely those of the authors and do not necessarily represent those of their affiliated organizations, or those of the publisher, the editors and the reviewers. Any product that may be evaluated in this article, or claim that may be made by its manufacturer, is not guaranteed or endorsed by the publisher.

## Supplementary Material

Supplementary Figure 1 The typical demonstration of No. 12 lymph node dissection during the D2 lymphadenectomy. **(A)** The complete of No. 12a lymph node dissection during a laparoscopic radical distal gastrectomy. **(B)** The complete of No. 12a, 12b, and 12p lymph nodes dissection during an open radical distal gastrectomy. Both surgical procedures were performed under the principle of D2 lymphadenectomy for lower-third GC.

## Abbreviations

GC, gastric cancer; TVI, therapeutic value index; PFS, progression-free survival; OS, overall survival; AJCC, American Joint Committee on Cancer; CT, computed tomography; SSI, surgical site infection, LOS, length of stay.
