# Supplementary figures and images for: Extensive Dissection at No. 12 Station During D2 Lymphadenectomy Improves Survival for Advanced Lower-Third Gastric Cancer: A Retrospective Study From a Single Center in Southern China
Source: Front Oncol. 2022 Jan 11;11:760963. doi: 10.3389/fonc.2021.760963 (PMC8787051; doi:10.3389/fonc.2021.760963)

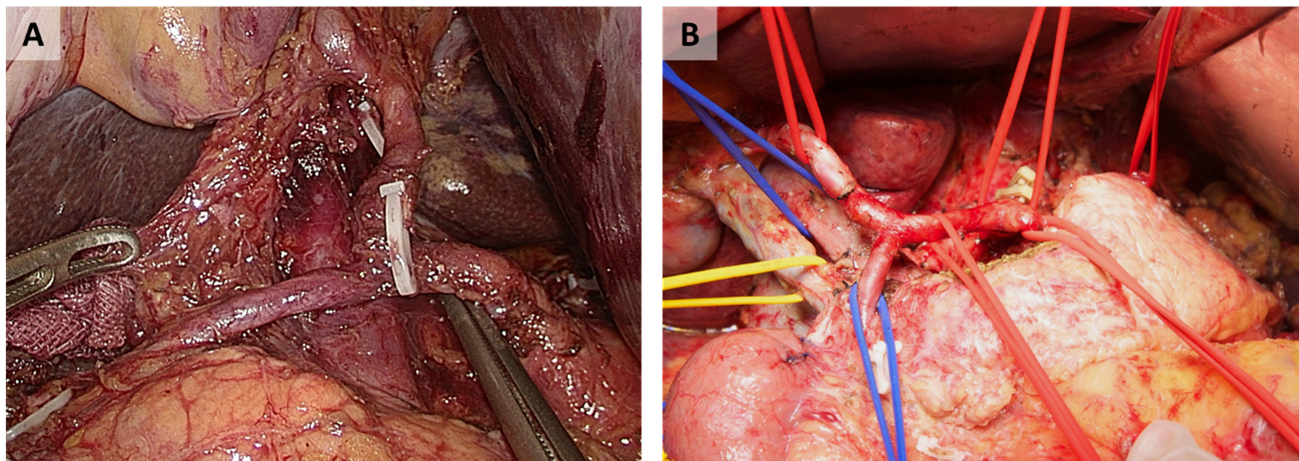

Supplement: Supplementary Figure 1 — The typical demonstration of No. 12 lymph node dissection during the D2 lymphadenectomy. (A) The complete of No. 12a lymph node dissection during a laparoscopic radical distal gastrectomy. (B) The complete of No. 12a, 12b, and 12p lymph nodes dissection during an open radical distal gastrectomy. Both surgical procedures were performed under the principle of D2 lymphadenectomy for lower-third GC. [file Image_1.png]
