# Supplementary material for: Extensive Dissection at No. 12 Station During D2 Lymphadenectomy Improves Survival for Advanced Lower-Third Gastric Cancer: A Retrospective Study From a Single Center in Southern China
Source: Front Oncol. 2022 Jan 11;11:760963. doi: 10.3389/fonc.2021.760963 (PMC8787051; doi:10.3389/fonc.2021.760963)
Supplement: Supplementary file 2 [file Table_1.docx]

| **Table S1.** The cumulative summary of harvested and metastatic lymph nodes from GC patients | | |
| --- | --- | --- |
|  | Control group  (n=174) | Study group  (n=179) |
|  | *D2 with 12a* | *D2 with 12a* ***+****12b,12p* |
| No. 1 |  |  |
| Harvested amount | 570 | 511 |
| Metastatic amount | 103 | 58 |
| LN metastasis ratio | 18.1 | 11.4 |
| No. 3 |  |  |
| Harvested amount | 936 | 900 |
| Metastatic amount | 290 | 188 |
| LN metastasis ratio | 31.0 | 20.9 |
| No. 4 |  |  |
| Harvested amount | 690 | 889 |
| Metastatic amount | 119 | 156 |
| LN metastasis ratio | 17.2 | 17.5 |
| No. 5 |  |  |
| Harvested amount | 424.0 | 426.0 |
| Metastatic amount | 118.0 | 106.0 |
| LN metastasis ratio | 27.8 | 24.9 |
| No. 6 |  |  |
| Harvested amount | 959 | 879 |
| Metastatic amount | 244 | 263 |
| LN metastasis ratio | 25.4 | 29.9 |
| No. 7 |  |  |
| Harvested amount | 752 | 791 |
| Metastatic amount | 143 | 95 |
| LN metastasis ratio | 19.0 | 12.0 |
| No. 8 |  |  |
| Harvested amount | 518 | 621 |
| Metastatic amount | 45 | 49 |
| LN metastasis ratio | 8.7 | 7.9 |
| No. 9 |  |  |
| Harvested amount | 209 | 281 |
| Metastatic amount | 34 | 18 |
| LN metastasis ratio | 16.3 | 6.4 |
| No. 11 |  |  |
| Harvested amount | 115 | 197 |
| Metastatic amount | 16 | 16 |
| LN metastasis ratio | 13.9 | 8.1 |
| No. 12 |  |  |
| Harvested amount | 377 | 804 |
| Metastatic amount | 19 | 25 |
| LN metastasis ratio | 5.0 | 3.1 |
| Overall LN stations |  |  |
| Harvested amount | 6039 | 6858 |
| Metastatic amount | 1235 | 1100 |
| LN metastasis ratio | 20.4 | 16.0 |
| Abbreviations: GC, gastric cancer; LN, lymph node. | | |

| **Table S2.** The long-term recurrence patterns after radical surgery for gastric cancer | | | |
| --- | --- | --- | --- |
| Pattern | Control group  (n=174) | Study group  (n=179) | *P value* |
|  | *D2 with 12a* | *D2 with 12a* ***+****12b,12p* |  |
| Locoregional recurrence, n (%) | 25 (14.4) | 23 (12.8) | 0.757 |
| Distant LN recurrence, n (%) | 85 (48.9) | 68 (38.0) | 0.042 |
| Peritoneal recurrence, n (%) | 78 (44.8) | 61 (34.1) | 0.039 |
| Hematogenous recurrence, n (%) | 35 (20.1) | 22 (12.3) | 0.029 |
| Liver metastasis | 15 (8.6) | 6(3.4) | 0.036 |
| Lung metastasis | 6 (3.4) | 5(2.8) | 0.723 |
| Brain metastasis | 0 (0) | 0 (0) | NA |
| Bone metastasis | 6 (3.4) | 5 (2.8) | 0.723 |
| The *chi*-square exact test used to compare differences between both groups. NA means no available value; LN means lymph node. | | | |
